# *TaBT1*, affecting starch synthesis and thousand kernel weight, underwent strong selection during wheat improvement

**DOI:** 10.1093/jxb/erz032

**Published:** 2019-02-07

**Authors:** Yamei Wang, Jian Hou, Hong Liu, Tian Li, Ke Wang, Chenyang Hao, Hongxia Liu, Xueyong Zhang

**Affiliations:** 1 Key Laboratory of Crop Gene Resources and Germplasm Enhancement, Institute of Crop Science, Chinese Academy of Agricultural Sciences (CAAS), Beijing, China; 2 Crop Genomics and Bioinformatics Center and National Key Lab of Crop Genetics and Germplasm Enhancement, College of Agricultural Sciences, Nanjing Agricultural University, Weigang, Nanjing, Jiangsu, China

**Keywords:** Haplotype, molecular marker, selection, starch synthesis, *TaBT1*, thousand kernel weight (TKW), wheat

## Abstract

BRITTLE1 (BT1), responsible for unidirectional transmembrane transport of ADP-glucose, plays a pivotal role in starch synthesis of cereal grain. In this study, we isolated three *TaBT1* homoeologous genes located on chromosomes 6A, 6B, and 6D in common wheat. *TaBT1* was mainly expressed in developing grains, and knockdown of *TaBT1* in common wheat produced a decrease in grain size, thousand kernel weight (TKW), and grain total starch content. High diversity was detected at the *TaBT1*-*6B* locus, with 24 polymorphic sites forming three haplotypes (*Hap1*, *Hap2*, and *Hap3*). Association analysis revealed that *Hap1* and *Hap2* were preferred haplotypes in modern breeding, for their significant correlations with higher TKW. Furthermore, β-glucuronidase (GUS) staining and enzyme activity assays in developing grains of transgenic rice with exogenous promoters indicated that the promoters of *Hap1* and *Hap2* showed stronger driving activity than that of *Hap3*. Evolutionary analysis revealed that *BT1* underwent strong selection during wheat polyploidization. In addition, the frequency distribution of *TaBT1*-*6B* haplotypes revealed that *Hap1* and *Hap2* were preferred in global modern wheat cultivars. Our findings suggest that *TaBT1* has an important effect on starch synthesis and TKW, and provide two valuable molecular markers for marker assisted selection (MAS) in wheat high-yield breeding.

## Teaser

Knockdown of *TaBT1* caused decline of wheat grain starch content, and natural variations in its promoter region led to significant differences in thousand kernel weight.

## Introduction

Common wheat (*Triticum aestivum* L.) is one of the most important staple crops worldwide, providing ~50% of human calorie intake. It has been estimated that wheat production needs to increase by 70% from the present level to meet the demand of an increased world population by 2050 (International Wheat Genome Sequencing Consortium, 2014). With reduction of arable land area, increasing yield is the foremost objective for world wheat breeding programs (Khush, 2013). Wheat production improvement in China mainly depends on the increase of yield per unit area, while thousand kernel weight (TKW) has contributed the most over the past six decades (Zheng *et al.*, 2011; Wang *et al.*, 2012; Zhang *et al.*, 2012). As grain filling directly determines grain weight and starchy endosperm comprises >80% of the cereals grain, increasing starch synthesis could effectively improve TKW and yield (Barron *et al.*, 2007; Zuo and Li, 2013).

Starch synthesis in wheat grain involves the participation of multiple enzymes and transporters (International Wheat Genome Sequencing Consortium, 2014; Kumar *et al.*, 2018). Sucrose, the primary substrate of starch, is first transported into the grain endosperm cells by the sucrose transporter (SUT) (Deol *et al.*, 2013). Then, under successive catalysis of sucrose synthase (SUS) and UDP-glucose pyrophosphorylase (UDPase), sucrose is converted into glucose-1-phosphate (G1P) (Neuhaus and Emes, 2000). After the catalysis of ADP-glucose pyrophosphorylase (AGPase), G1P is converted into ADP-glucose (ADP-Glc), the precursor of starch. Then ADP-Glc is transported into amyloplast for starch synthesis by the BT1 protein located on its membrane (Beckles *et al.*, 2001). As 65–95% of the total AGPase activity in wheat endosperm is cytoplasmic (Tetlow *et al.*, 2003), this implies that most of the ADP-Glc for starch synthesis is synthesized in the cytoplasm. Therefore, BT1 protein, responsible for transmembrane transportation of ADP-Glc, plays a vital role in starch synthesis (Denyer *et al.*, 1996; Thorbjørnsen *et al.*, 1996; Burton *et al.*, 2002).

BT1 belongs to the mitochondrial carrier family (MCF), which is responsible for the translocation of small molecules between the mitochondria and cytoplasm (Haferkamp, 2007; Palmieri and Monné, 2016). Generally, MCF proteins are presumed to be located in the inner membrane of mitochondria (Millar and Heazlewood, 2003); some are believed to be involved in the transport of multiple solutes across organelle membranes (Leroch *et al.*, 2005; Palmieri *et al.*, 2006; Kirchberger *et al.*, 2008). The *bt1* mutant was first discovered in maize, which shows reduced rates of ADP-Glc uptake into amyloplasts and accumulates substantial ADP-Glc in the cytoplasm, leading to a collapsed angular appearance of amyloplasts at maturity (Mangelsdorf, 1926; Liu *et al.*, 1992; Tobias *et al.*, 1992; Shannon *et al.*, 1996, 1998). Rice *OsBT1* could also affect starch synthesis and starch granule formation, and its mutants result in smaller grain size and starch granules (Cakir *et al.*, 2016; Li *et al.*, 2017). In barley, maize, and wheat, BT1 maintains a conserved function in importing ADP-Glc by counter-exchange with ADP *in vitro* (Bowsher *et al.*, 2007; Kirchberger *et al.*, 2007; Soliman *et al.*, 2014).

To date, studies on the physiological role of wheat BT1 protein have seldom been reported. The aim of the current study was to examine the phenotypic effect of wheat *BT1*, identify its favorable alleles, and investigate the breeding selection to *TaBT1*. In order to address these questions, we first discuss the alteration of starch granule morphology, starch content, and yield-related components in RNAi transgenic wheat. Secondly, we conducted association analysis between *TaBT1* haplotypes and yield-related phenotypes. Thirdly, we evaluated the frequency of *TaBT1* haplotypes in wheat modern cultivars and its nucleotide diversity in wheat wild relatives. Our results demonstrate the essential role of *BT1* in wheat starch synthesis and TKW, and its potential for wheat molecular breeding.

## Materials and methods

### Plant materials and field trials

Two populations, Chinese 157 landraces from the mini-core collection (MCC) and 348 Chinese modern cultivars (MC) released since the 1940s (see Supplementary Table S1 at *JXB* online), were used for association analysis between genotypes and yield-related phenotypes (Hao *et al.*, 2011). Two populations were planted at Luoyang, Henan province (China) during 2002–2003 (2002 LY) and 2005–2006 (2005 LY) cropping seasons, and at Shunyi, Beijing (China) in the 2010–2011 (2010 SY) cropping season. Field trials were conducted in randomized complete blocks with three replicates at all locations. Each plot consisted of three 2 m rows spaced 20 cm apart. Collection of phenotypic traits was followed as described by Zheng *et al.* (2014). Ten plants from the middle of each plot were randomly sampled. Phenotypic traits include effective tiller number (ETN), spikelet number per spike (SN), grain number per spike (GN), TKW, kernel length (KL), kernel width (KW), and kernel thickness (KT).

A wheat introgression line derived from Fumai/Handan 6172 (BC_3_F_7_) (Supplementary Table S2) was used to verify the effect of *TaBT1*-*6B* haplotypes on TKW. Fifteen common wheat cultivars (Supplementary Table S3) were used for expression difference analysis among *TaBT1*-*6B* haplotypes. A total of 67 wheat ancestor collections (Supplementary Table S4) were used in an evolutionary study of *BT1* homoeologous genes. These collections comprised 17 diploid collections with the AA genome, 13 with the SS genome, 19 with the DD genome, and 18 tetraploid wheat collections with the AABB genome. In addition, Chinese 348 MC and 957 MC (Supplementary Table S5) from the other five major wheat production regions worldwide were used to investigate the global geographic distribution of *TaBT1*-*6B* haplotypes. These latter cultivars comprise 398 from North America, 53 from CIMMYT, 375 from Europe, 71 from the former USSR, and 60 from Australia.

### Cloning and sequencing of *TaBT1* homoeologous genes

All primers used in this study (Supplementary Table S6) were designed by the Primer Premier 5.0 software (http://www.premierbiosoft.com/; last accessed Jan 2019) and synthesized by the BGI Tech (Shenzhen, China). PCR was performed in a total volume of 15 μl, containing 7.5 μl of GC buffer I, 50 ng of DNA, 1 μl of forward and reverse primers (10 mM), 0.24 μl of dNTPs (25 mM), and 0.15 μl of LA *Taq* polymerase (Takara, Dalian). PCR was carried out in a Veriti 96-Well Thermal Cycler (Applied Biosystems, USA) with the following procedure: denaturing at 94 °C for 5 min, followed by 35 cycles of 94 °C for 30 s, annealing at 55–65 °C for 45 s, and 72 °C for extension (1 kb min^–1^), and finally extension at 72 °C for 10 min. PCR products were purified by an AxyPrep DNA Gel Extraction Kit (Axygen Biosciences, Hangzhou), cloned into the *pEASY*-T1 Cloning Vector, and then transformed into *Trans*1-T1 competent cells by the heat shock method (TransGen Biotech, Beijing). Conjugative plasmids were extracted with an AxyPrep Plasmid Miniprep Kit (Axygen Biosciences) and sequenced by an ABI 3730XI DNA Analyser (Applied Biosystems).

### Gene expression analysis

For assay of the spatial and temporal expression pattern of *TaBT1*, various tissue samples were collected at different developmental stages at 06.00 h from *T*. *aestivum* cv. Chinese Spring grown in the field at the Chinese Academy of Agricultural Sciences (CAAS) experimental station in Beijing during the natural cropping season in 2014–2015. For assay of the rhythmic expression pattern of *TaBT1*, flag leaves and developing grains at 10 days post-anthesis (DPA) were sampled from Chinese Spring in a growth chamber simulating the natural light intensity in the field with light–dark cycle conditions of 12 h light/12 h dark at 20 °C. The samples were collected in two cycles from 06.00 h and subsequently every 4 h.

Total RNA was extracted with a RNAprep Pure Plant Kit Plus Reagent (TIANGEN Biotech, Beijing), and cDNA was synthesized with the FastQuant RT Kit (TIANGEN Biotech). Gene expression was measured by quantitative real-time PCR (qRT-PCR) in a total volume of 20 μl, containing 2 μl of cDNA, 0.4 μl of each primer (2 μM), 0.4 μl of ROX Reference Dye (50×), and 10 μl of 2× SYBR^®^ Premix Ex Taq (Takara, Dalian). *TaActin* was used as the endogenous control to normalize expression levels of different samples. All assays were performed and analyzed from two independent experiments, each consisting of three technical repetitions.

### Subcellular localization

To investigate the subcellular localization of *TaBT1*, the cDNA sequence of *TaBT1*-*6D* without the termination codon was isolated from Chinese Spring with primer set Sub-F containing a *Hind*III site and Sub-R with a *Bam*HI site (Supplementary Table S6). After sequencing verification, the fragment was digested with *Hind*III and *Bam*HI, and fused to the green fluorescent protein (GFP) gene in the pJIT163-GFP vector to generate *TaBT1*–GFP. The fusion construct and GFP control were each introduced into wheat protoplasts from seeding leaves following Mao *et al.* (2011). After an incubation at 22 °C for 18 h in the dark, the fluorescence signal of GFP was observed under a confocal laser scanning microscope (LSM880, Carl Zeiss, Germany). At least 10 independent protoplasts were imaged per experiment.

### Wheat transformation and Southern blotting

A trigger fragment of 355 bp derived from the coding sequence of *TaBT1-6D* was used to generate the *TaBT1*-RNAi construct. The sense fragment was amplified with primer pair BT1-147F containing a *Bam*HI site and BT1-501R with a *Kpn*I site. The antisense fragment was amplified with primer pair BT1-A147F containing a *Sac*I site and BT1-A501R with a *Spe*I site. After the two fragments were successively cloned to pWMB006 vector and ascertained as the correct sequence, the resulting construct was digested by *Hind*III and *Eco*RI, and cloned into pCAMBIA3301 vector. The *TaBT1*-RNAi construct was first mobilized into *Agrobacterium tumefaciens* strain EHA105, and then transformed into *T*. *aestivum* cv. Fielder by the *A. tumefaciens*-mediated transformation method (Wang *et al.*, 2017). DNA of the transformed plants was extracted by the cetyltrimethylammonium bromide (CTAB) method (Doyle, 1987). The positive lines were verified by PCR amplification with primer pair BT1-A501R/NosR2 (Supplementary Table S6).

Southern blotting was conducted as described by Wang *et al.* (2017). In brief, a large amount of total genomic DNA was first extracted with a standard CTAB method (Sambrook *et al.*, 1989); then 10 μg of DNA from each sample was digested with *Hind*Ш. The digested DNA were then fractionated on a 0.8% agarose gel and transferred onto a nylon Hybond-N^+^ membrane. PCR products of the *bar* gene (429 bp), amplified by bar-F/R, were labeled with digoxigenin and used as probes to hybridize with the digested DNA on the membrane. The hybridization and detection steps were conducted with the DIG High Prime DNA Labeling and Detection Starter Kit II (Roche, Germany).

### Phenotypic identification of *TaBT1*-RNAi transgenic wheat

Both the wild-type control and transgenic plants were grown in the field at the CAAS experimental station in Beijing (40.18°N, 116.58°E) during the natural growing season. Positive transgenic lines were self-pollinated and harvested, and transgenic T_2_ plants were used for further analysis. Developmental grains at 10 DPA was sampled for *TaBT1* expression assay, and *TaActin* was used as the internal control. For each line, three biological replicates, each with three technical replicates, were performed and the data were expressed as means ±SE. At maturity, SN and GN were evaluated from main stem spikes. After grains from single plants were harvested and dried, KL, KW, and KT of each plant were measured following the method described by Qin *et al.* (2014), while TKWs were converted from grain weight of an individual plant. Phenotypic data of each line came from the average value of 15 individual plants.

For the observation of starch granules, mature grains of wild-type and transgenic wheat were crushed with a mortar, and the surface of ruptured transverse sections was coated with gold powder to prepare samples for observations. All samples were then observed by field emission SEM (SU8010, Hitachi, Japan). At least two mature grains from three independent plants of each line were imaged. Micrographs of each sample were taken with a magnification of ×600. Based on this, the starch granule number of each line was counted from five micrographs of the same size. Grain total starch content was quantified according to the national standard (NY/T11-1985) for cereals with the optical rotation method. Data were collected from at least five plants with three replicates per line.

### SNP detection and molecular marker development

Thirty-six common wheat cultivars with high diversity (Supplementary Table S7) were used to detect the diversity of *TaBT1* homoeologous genes. After sequencing, the sequences were aligned by DNAMAN (http://www.lynnon.com/; last accessed Jan 2019), and SNPs (single nucleotide polymorphisms) were identified by DNASTAR (http://www.dnastar.com/; last accessed Jan 2019). Two molecular markers were developed to discriminate the three haplotypes at *TaBT1*-*6B*. Compared with *Hap2* and *Hap3*, *Hap1* has a deletion of 4 bp at base pair position –2029. Based on this, the *InDel-2029* marker was developed by two-step PCR. In the first round, the genome-specific primer set B-2F/4R was used to amplify fragments in all cultivars. Then the PCR product was diluted 30 times, and 1 μl was taken as template for the second round PCR with primers B-357F/569R. After the two-step PCR, the fragment length of *Hap1* was 192 bp, whereas those of *Hap2* and *Hap3* were 196 bp. The *CAPS-1664* marker was developed based on the SNP (T/C) at base pair position –1664. Only the amplified fragments of *Hap3* could be cleaved by the restriction endonuclease *Acl*I. Thus, the PCR product of *Hap1* and *Hap2* amplified by the genome-specific primer pair B-CAPS-F/R was 1099 bp, whereas those of *Hap3* were 778 bp and 321 bp after digestion.

### Rice transformation and β-glucuronidase (GUS) activity assay

The promoter region (~2.5 kb) of three haplotypes at *TaBT1-6B* were isolated with primers Pro-Fe and Pro-Re, introducing DNA restriction enzyme sites *Hind*III and *Eco*RI. The three fragments were then inserted into vector pCAMBIA1391Z containing the *GUS* reporter gene; pCAMBIA1391Z with its native promoter was used as the vector control. Constructs were first mobilized into *A*. *tumefaciens* strain EHA105, and then transformed into *Oryza sativa* cv. Kitaake by the *A. tumefaciens*-mediated transformation method (Hiei *et al.*, 1994). Positive transgenic rice lines of the T_1_ generation were grown at Langfang (39.03°N, 116.04°E), Hebei Province (China), and grains at 21 DPA were sampled for subsequent assays.

GUS staining was performed as described by Kosugi *et al.* (1990). Images of stained grains were captured by using a Discovery V20 stereomicroscope (Carl Zeiss, Germany). Fluorometric GUS activity assays were based on a previous method with minor modifications (Hänsch *et al.*, 1995). For each sample, 200 mg of fresh grains were ground into powder after freezing in liquid nitrogen, and then homogenized in GUS extraction buffer (0.02885 M Na_2_HPO_4_, 0.02115 M NaH_2_PO_4_, 0.01 M EDTA, 0.1% SDS, 0.1% β-mercaptoethanol, and Triton X-100). The homogenate was then centrifuged at 12 000 rpm for 10 min at 4 °C. Protein concentration in the supernatant was measured according to the method described by Bradford (1976). The supernatant was assayed for GUS activity with 4-methylumbellifery-β-d-glucuronic acid (4-MUG) as the substrate. Fluorescence values were measured on a TriStar LB941 fluorescence spectrophotometer (Berthold, Germany) with 4-methylumbelliferon (4-MU) as the calibration control.

### Statistical analysis

One-way ANOVA was performed using SPSS Statistics 17.0 (http://www-01.ibm.com/software/analytics/spss/; last accessed Jan 2019). Tukey’s multiple test was employed for multiple comparisons (*P<0.05, **P<0.01, and ***P<0.001). *Cis*-elements were predicted by PLACE (http://www.dna.affrc.go.jp/PLACE/; last accessed Jan 2019) and Plantcare (http://bioinformatics.psb.ugent.be/webtools/plantcare/html/; last accessed Jan 2019). Nucleotide diversity analysis and Tajima’s *D*-tests were carried out by DnaSP 5.10 (http://www.ub.edu/dnasp/; last accessed Jan 2019). Genetic differentiation among populations was estimated by the *F*_ST_ index with Arleqiun 3.5.1.2 (http://cmpg.unibe.ch/software/arlequin3/; last accessed Jan 2019).

## Results

### Isolation and sequence analysis of *TaBT1* homoeologous genes

**Fig. 1.**
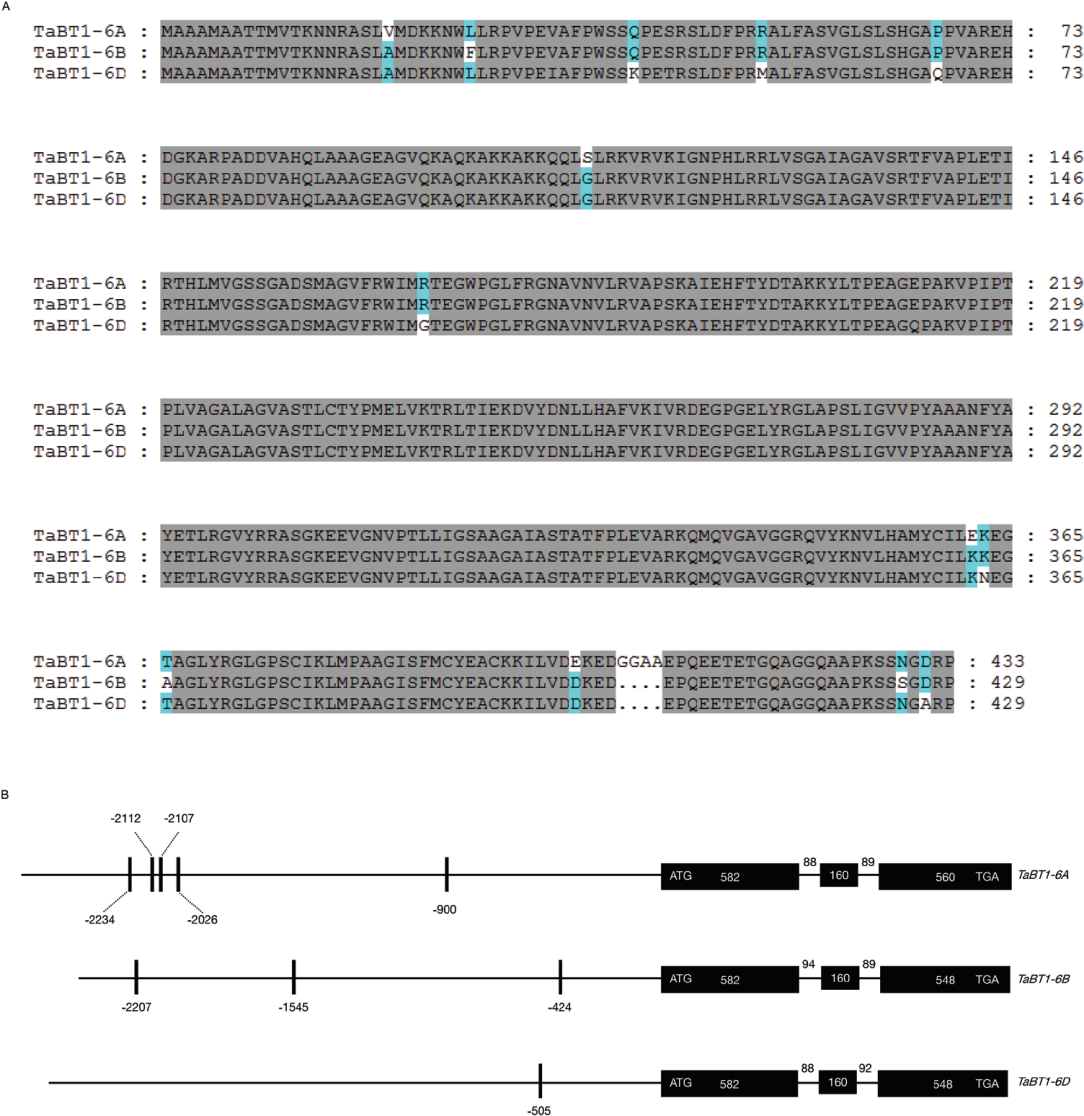
Sequence alignment and gene structures of *TaBT1-6A*, *TaBT1-6B*, and *TaBT1-6D*. (A) Protein sequence alignments of *TaBT1-6A*, *-6B*, and *-6D*. (B) Gene structures of *TaBT1-6A*, *-6B*, and *-6D*. Black rectangles represent exons, horizontal lines between exons signify introns, horizontal lines on the left of the first exons represent promoter regions, vertical lines signify the position of an Skn1 motif, and numbers denote size (bp).

Primers *TaBT1*-5F/1536R based on the mRNA sequence of *TaBT1* (GenBank accession no. BT008958.1) were designed to amplify the genomic DNA and cDNA from Chinese Spring. All three homoeologous genes consist of three exons and two introns, and the genomic sequence lengths of *TaBT1*-*6A*, -*6B*, and -*6D* are 1479, 1473, and 1470 bp, respectively. The coding sequences of both *TaBT1*-*6B* and -*6D* are 1290 bp, encoding 430 amino acid polypeptides, whereas that of *TaBT1*-*6A* is 1302 bp, encoding a 434 amino acid polypeptide. Moreover, the proteins encoded by the three homoeologous genes show a high identity of >98% (Fig. 1A). In addition, the promoter regions of *TaBT1*-*6A*/*6B*/*6D* (~2.5 kb) were obtained from wheat 3BSEQ (http://wheat-urgi.versailles.inra.fr/Projects/3BSeq; last accessed Jan 2019), and a Skn-1 motif in the promoters of all the three genomes was predicted to be relevant to endosperm expression (Fig. 1B). By aligning to the genomic sequence of Chinese Spring (International Wheat Genome Sequencing Consortium, 2014), three homoeologous genes are physically mapped to the 189.92 Mb position on chromosome 6A, 276.74 Mb on 6B, and 152.27 Mb on 6D.

### *TaBT1* is mainly expressed in developing grains and is localized to the plastid

**Fig. 2.**
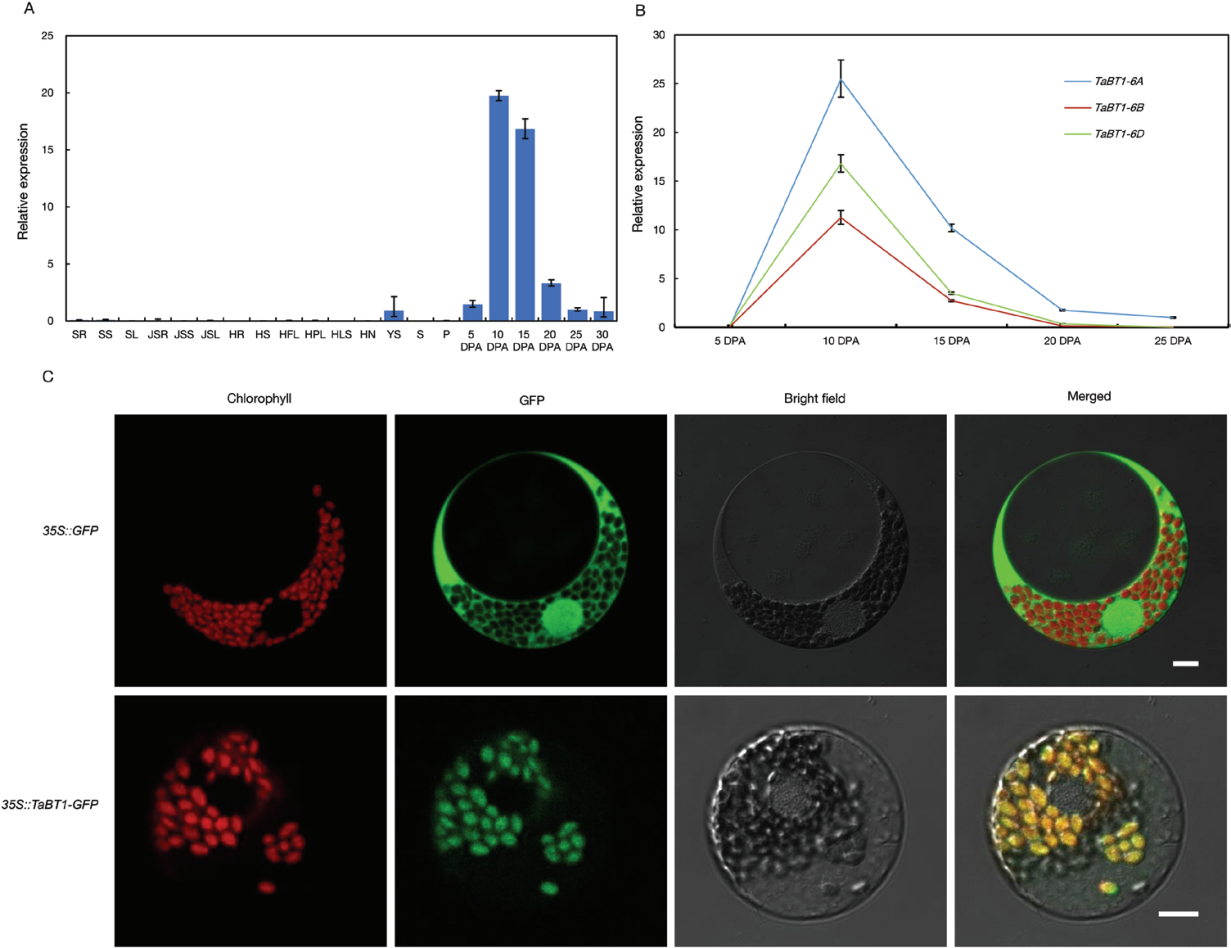
*TaBT1* is mainly expressed in tender developing grains and is localized to the chloroplast of wheat protoplast. (A) Mean relative expression of *TaBT1* in different tissues of Chinese Spring. SR, seedling roots; SS, seedling stems; SL, seedling leaves; JSR, roots at the jointing stage; JSS, stems at the jointing stage; JSL, leaves at the jointing stage; HR, roots at the heading stage; HS, stems at the heading stage; HFL, flag leaves at the heading stage; HPL, penultimate leaves at the heading stage; HLS, leaf sheaths at the heading stage; HN, nodes at the heading stage; YS, young spikes; S, stamens; P, pistils; 5 DPA, 10 DPA, 15 DPA, 20 DPA, 25 DPA, 30 DPA, grains at different developmental stages, namely 5, 10, 15, 20, 25, and 30 DPA. Expression of *TaBT1* in grains at 25 DPA was assumed to be 1. (B) Relative expression of *TaBT1* homoeologous genes in grains at different developmental stages. Expression of *TaBT1*-*6A* in grains at 25 DPA was assumed to be 1. (C) Subcellular localization of *TaBT1*. *GFP* and *TaBT1*–*GFP* fusions under the control of the *Cauliflower mosaic virus* 35S promoter were transiently expressed in wheat proplasts. Eighteen hours after transformation, the fluorescence signal of GFP was observed under a confocal laser scanning microscope. Chlorophyll autoﬂuorescence (red), GFP (green), bright-field images, and an overlay of the merged ﬂuorescence (orange) are shown in each panel. Scale bar=10 μm.

qRT-PCR was employed to analyze the expression pattern of *TaBT1* in different tissues with the universal primer set BT1-RT-635F/881R. In the current study, *TaBT1* was mainly expressed in developing grains, and the transcription levels were extremely high from 10 to 15 DPA (Fig. 2A). As all tissues samples were collected at 06.00 h, to eliminate the effect of sampling time, we used flag leaves and developing grains of Chinese Spring at 10 DPA to study the circadian expression pattern of *TaBT1*. The results showed that *TaBT1* was highly expressed in developing grains after being exposed to darkness for 12 h, but no expression was detected in flag leaves throughout the cycle (Supplementary Fig. S1). This is a further confirmation that *TaBT1* is mainly expressed in developing grains. Then we analyzed the expression differences among *TaBT1*-*6A*, -*6B*, and -*6D* in developing grains with genome-specific qRT-PCR primers. As expected, the three homoeologous genes displayed similar expression patterns, but the expression levels differed: *TaBT1*-*6A* exhibited the highest level, -*6D* was slightly lower, and -*6B* was substantially lower at 10 and 15 DPA (Fig. 2B).

To investigate the subcellular localization of *TaBT1*, a *TaBT1*–GFP fusion was constructed and transiently expressed in wheat protoplasts. As shown in Fig. 2C, the free GFP fluorescence was observed in the entire cell, whereas *TaBT1*–GFP fusion protein fluorescence was targeted to the chloroplast, indicating that *TaBT1* is a plastid-localized protein.

### Knockdown of *TaBT1* induces alteration of grain weight and starch content

**Fig. 3.**
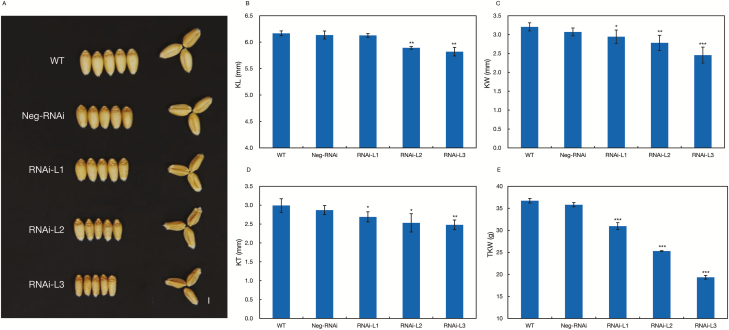
RNAi of *TaBT1* caused significant shrinkage of the mature grain. (A) Grain morphology; (B) kernel length, KL; (C) kernel width, KW; (D) kernel thickness, KT; (E) TKW. Data are means ±SD of 15 plants, and asterisks indicate significant differences between *TaBT1-*RNAi lines and wild-type plants; *P<0.05, **P<0.01, ***P<0.001 (*t*-test). Scale bar=2 mm.

To investigate the potential function of *TaBT1*, we constructed *TaBT1* transgenic RNAi lines in *T*. *aestivum* cv. Fielder. The 355 bp trigger fragment from *TaBT1-6D* for the RNAi vector construction shared 97.7% and 98.6% sequence similarity with *TaBT1-6A* and *TaBT1-6B*, respectively. A total of 24 individuals (T_0_) were obtained, of which 17 were transgenic positive lines, while the other 7 were negative. Compared with the wild type, the positive transgenic lines did not show any obvious alteration in ETN, SN, and GN (Supplementary Fig. S2), while a decrease in grain size was observed (Fig. 3A). Three representative field-grown positive lines with a double copy (Supplementary Fig. S3) had reductions by 0.7, 4.5, and 5.6% for KL (Fig. 3B), 8.1, 13.2, and 23.3% for KW (Fig. 3C), and 10.1, 15.4, and 17.1% for KT (Fig. 3D). Moreover, TKW in the three lines was significantly reduced by 15.7, 31.1, and 47.3%, respectively (Fig. 3E).

**Fig. 4.**
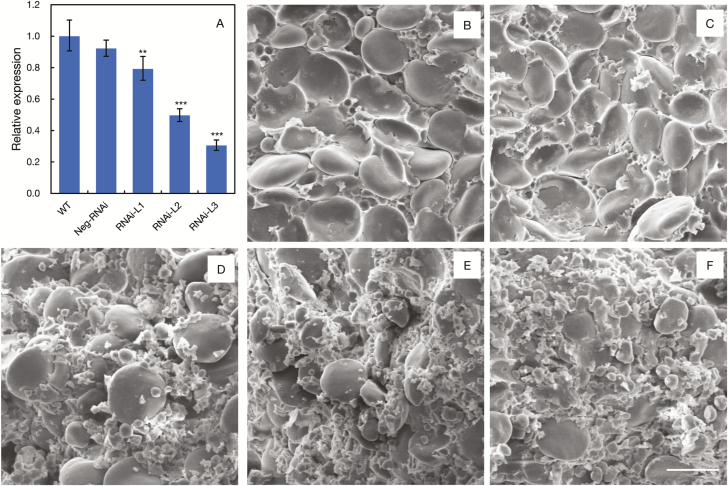
Starch granules in grain endosperms of *TaBT1* transgenic RNAi lines. (A) Expression levels of *TaBT1* in grains at 10 DPA from transgenic lines and control plants; grain starch granule morphology of (B) wild-type Fielder; (C) negative control lines; and positive lines of RNAi-L1 (D), RNAi-L2 (E), and RNAi-L3 (F); **P<0.01, ***P<0.001 (*t*-test). Scale bar=25 μm.

**Fig. 5.**
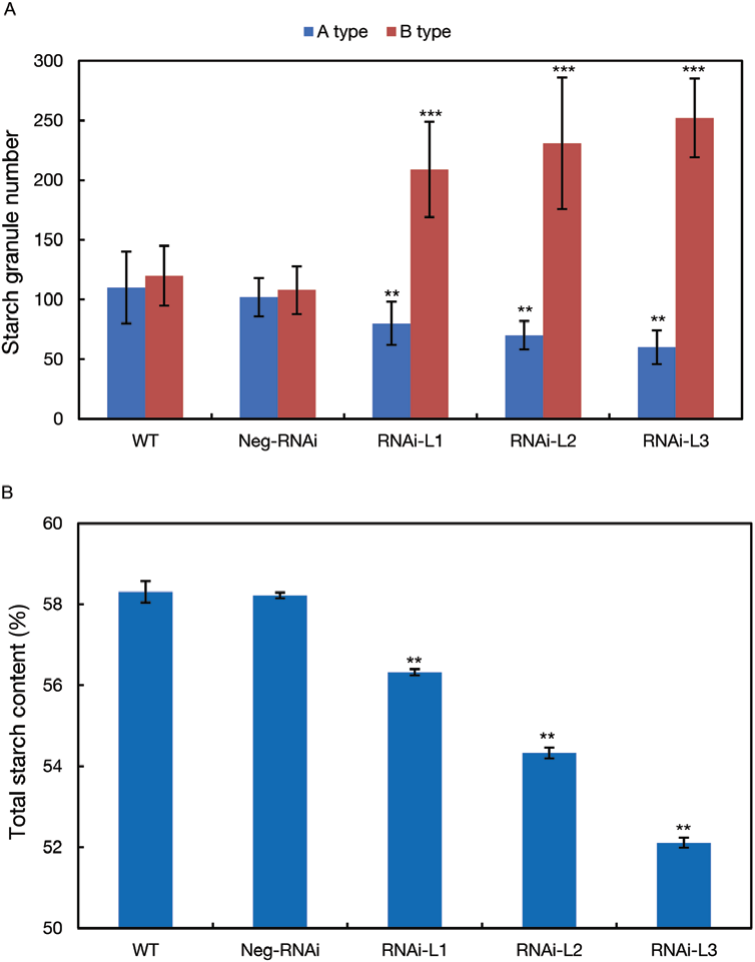
RNAi of *TaBT1* altered starch granule number and grain total starch content. (A) The number of starch granules; (B) starch content. **P<0.01, ***P<0.001 (*t*-test).

Expression analysis showed that compared with the wild type, transcript levels of *TaBT1* in the above three lines were reduced by 20.8, 50.3, and 69.5%, respectively (Fig. 4A). Further, all three homoeologous genes were simultaneously silenced in the transgenic lines (Supplementary Fig. S4). Also, the arrangement of starch granules became loose (Fig. 4B–F) and the proportion of A- and B-type starch granules was clearly changed (Fig. 5A). Compared with the wild type, the number of A-type starch granules in the three positive lines decreased by 27.3, 36.4, and 45.5%, respectively. In contrast, the number of B-type granules increased by 0.7, 0.9, and 1.1 times. Moreover, total starch contents in the three lines were reduced by 3.4, 6.8, and 10.6%, respectively (Fig. 5B). The above results reflect that *TaBT1* affects starch synthesis and TKW in wheat.

### Diversity is detected only at the *TaBT1*-*6B* locus in common wheat

**Fig. 6.**
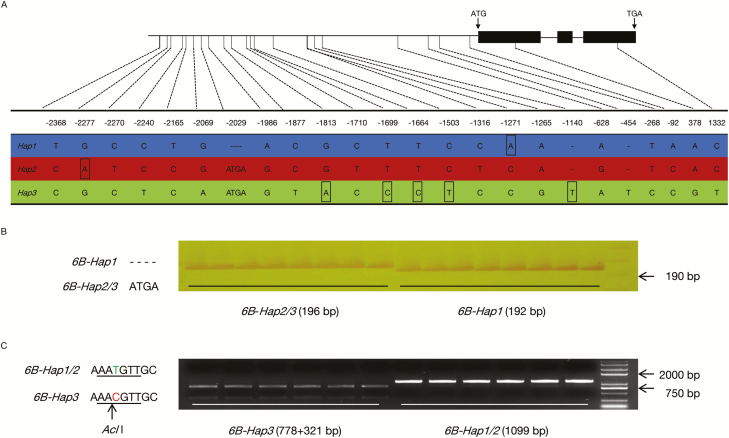
Haplotypes at *TaBT1-6B* and the molecular marker development based on its polymorphism. (A) Polymorphic sites at *TaBT1-6B*. Vertical lines indicate sites of variation; numbers represent corresponding positions (bp); black rectangles represent loci with haplotype-specific *cis*-elements. (B) The *InDel-2029* marker was based on the InDel at base pair –2029; cultivars with different haplotypes were discriminated on 6% denaturing polyacrylamide gels. (C) The *CAPS-1664* marker was designed for the SNP at base pair –1664; the black line and arrow represent recognition sites of the restriction endonuclease *Acl*I.

Considering the obvious effects of *TaBT1* in wheat, we then detected its nucleotide polymorphism with 36 common wheat cultivars with high diversity (Supplementary Table S7) to find preferred alleles that can be used in wheat production. No allelic variation was found at either *TaBT1*-*6A* or *TaBT1*-*6D* loci, whereas 24 polymorphic sites were detected at *TaBT1*-*6B*, with two SNPs (base pair 378 A/G; base pair 1332 C/T) falling within the coding region and the rest in the promoter region. These variations formed three haplotypes, *Hap1*, *Hap2*, and *Hap3*. Moreover, haplotype-specific *cis*-elements at the mutation sites in the promoter region were predicted (Fig. 6A). Two molecular markers, named *InDel-2029* and *CAPS-1664*, were developed to discriminate the three haplotypes (Fig. 6B, C). Based on a deletion of 4 bp at base pair position –2029 (Fig. 6A), the *InDel-2029* marker was developed to discriminate *Hap1* from *Hap2* and *Hap3*. After two-step amplification, the PCR products could be distinguished on 6% denaturing polyacrylamide gels (Fig. 6B). The *CAPS-1664* marker was developed based on the SNP (T/C) at base pair –1664 (Fig. 6A). After being cleaved by the restriction endonuclease *Acl*I, the amplified fragments of *Hap3* could be discriminated from *Hap1* and *Hap2* (Fig. 6C).

### Strong haplotype effect on TKW is detected at *TaBT1-6B*

The *InDel-2029* and *CAPS-1664* markers were used to genotype 157 landraces from the MCC and 348 cultivars from the MC. Differences in ETN, SN, GN, KL, KW, and KT were not notable among *TaBT1*-*6B* haplotypes, while the differences in TKW were significant (Table 1). In landraces, the mean TKW of *Hap1* was significantly higher than that of *Hap3* by 22.8% in 2005 (*P*<0.01). In modern cultivars, the mean TKW of *Hap1* was significantly higher than that of *Hap3* in all three environments (*P*<0.01) by 19.2% in 2002 LY, 20.9% in 2005 LY, and 12.3% in 2010 SY, respectively. In addition, the mean TKW of *Hap2* was markedly higher than that of *Hap3* by 13.4% in 2002 LY and 16.0% in 2005 LY (*P*<0.01). However, no significant difference was found between *Hap1* and *Hap2* in landraces or modern cultivars. These findings show that *Hap1* and *Hap2* are preferred haplotypes correlated with higher TKW. Additionally, the haplotype effects on TKW between *Hap1* and *Hap3* were proved in a wheat introgression line derived from Fumai/Handan 6172; the mean TKW of *Hap1* (50.6 g) was significantly higher than that of *Hap3* (45.3 g) at *P*=0.05 (Supplementary Table S2). These results indicate that *TaBT1-6B-Hap1* and *-Hap2* positively regulate TKW.

**Table 1.** Comparisons on yield-related traits among *TaBT1-6B* haplotypes in the Chinese mini-core collection (MCC) and modern cultivars (MC) grown in three environments

	2002 LY			2005 LY			2010 SY		
	*Hap1*	*Hap2*	*Hap3*	*Hap1*	*Hap2*	*Hap3*	*Hap1*	*Hap2*	*Hap3*
**MCC**									
ETN	9.50±0.94 a	9.09±0.44 a	8.29±0.42 a	9.78±1.02 a	8.73±0.40 a	8.81±0.38 a	14.59±1.02 a	15.60±0.60 a	14.87±0.51 a
SN	20.78±1.28 a	21.64±0.32 a	21.70±0.30 a	20.98±0.61 a	20.83±0.20 a	20.70±0.23 a	20.63±0.70 a	21.18±0.21 a	20.06±0.23 a
GN	51.67±4.76 a	50.04±1.56 a	50.96±1.34 a	40.67±3.53 a	40.91±0.91 a	40.25±0.89 a	49.40±3.51 a	51.44±1.16 a	53.57±1.05 a
**TKW (g)**	**35.80±1.62 a**	**35.17±1.19 a**	**32.64±0.73 a**	**35.72±2.28 a**	**31.63±0.88 a b**	**29.09±0.61 b**	**33.12±1.17 a**	**33.23±0.84 a**	**31.38±0.65 a**
KL (mm)	0.64±0.01 a	0.64±0.01 a	0.62±0.01 a	0.65±0.01 a	0.64±0.01 a	0.62±0.01 a	0.67±0.01 a	0.66±0.01 a	0.65±0.01 a
KW (mm)	0.30±0.01 a	0.30±0.00 a	0.29±0.00 a	0.31±0.01 a	0.30±0.00 a	0.29±0.00 a	0.31±0.01 a	0.30±0.00 a	0.30±0.00 a
KT (mm)	0.28±0.00 a	0.26±0.00 a	0.26±0.00 a	0.28±0.01 a	0.27±0.00 a	0.27±0.00 a	0.29±0.00 a	0.29±0.00 a	0.29±0.00 a
**MC**									
ETN	7.05±0.22 a	7.24±0.24 a	7.95±0.54 a	8.77±0.28 a	9.12±0.30 a	10.05±0.73 a	11.39±0.26 a	11.84±0.34 a,b	13.57±0.66 b
SN	21.38±0.22 a	21.16±0.25 a	21.15±0.34 a	21.18±0.16 a	21.34±0.18 a	21.32±0.40 a	20.56±0.15 a	20.88±0.16 a	20.50±0.38 a
GN	52.39±0.93 a	52.66±1.02 a	51.24±2.01 a	46.51±0.70 a	47.64±0.79 a	47.48±1.25 a	53.50±0.50 a	54.65±0.74 a	50.46±1.71 a
**TKW (g)**	**44.19±0.51 a**	**42.04±0.64 a**	**37.07±1.06 b**	**40.91±0.48 a**	**39.28±0.58 a**	**33.86±1.06 b**	**40.71±0.51 a**	**39.75±0.53 a,b**	**36.24±0.99**
KL (mm)	0.68±0.00 a	0.67±0.00 a	0.66±0.01 a	0.69±0.00 a	0.69±0.00 a	0.66±0.01 b	0.69±0.00 a	0.69±0.00 a	0.66±0.01 b
KW (mm)	0.34±0.00 a	0.33±0.00 a	0.33±0.00 a	0.33±0.00 a	0.33±0.00 a	0.31±0.00 b	0.33±0.00 a	0.32±0.00 a	0.32±0.00 a
KT (mm)	0.29±0.00 a	0.29±0.00 a	0.28±0.00 a	0.29±0.00 a	0.29±0.00 a,b	0.28±0.00 b	0.30±0.00 a	0.31±0.01 a	0.29±0.00 a

ETN, effective tiller number; SN, spike number per spike; GN, grain number; TKW, thousand kernel weight; KL, kernel length; KW, kernel width; KT, kernel thickness; 2002 LY, Luoyang (2002); 2005 LY, Luoyang (2005); 2010 SY, Shunyi (2010).

Different lower case letters indicate significant differences between haplotypes at *P*<0.01.

### Expression differences among *TaBT1*-*6B* haplotypes are mainly attributed to variations in the promoter region

**Fig. 7.**
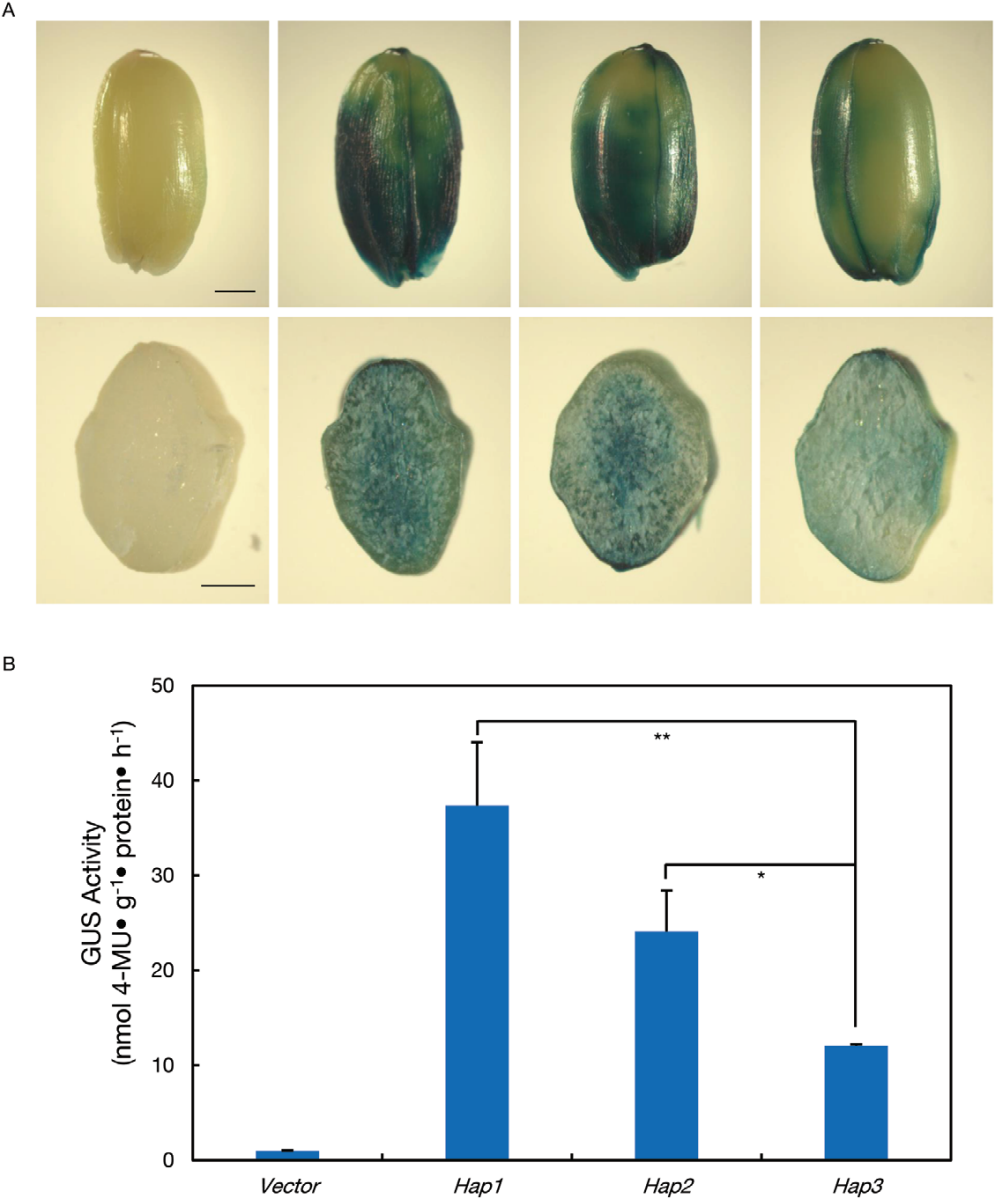
Histochemical and fluorometric GUS assays in transgenic rice grains at 21 DPA indicated that the promoters of *Hap1* and *Hap2* at *TaBT1-6B* possess stronger driving ability than that of *Hap3*. (A) GUS staining of transgenic rice grains. (B) β-Glucuronidase activities in transgenic rice grains. *P<0.05, **P<0.01 (*t*-test). Scale bar=500 μm.

To explore the possible causes of haplotype effects, we further investigated the sequence differences among *TaBT1*-*6B* haplotypes. Since variations in the coding region did not lead to changes in amino acids, we then focused on polymorphisms in the promoter region. Thus, the promoters of *Hap1*, *Hap2*, and *Hap3* were each integrated into the vector pCAMBIA1391Z to drive *GUS* gene expression in transgenic rice (Fig. 7). Histochemical staining showed that developing grains with exogenous promoters of *Hap1* and *Hap2* were stained distinctly darker than those of *Hap3*, while grains carrying the vector control were not stained (Fig. 7A). In addition, the GUS activities in grains of *Hap1* and *Hap2* were 3.1 and 2.0 times significantly higher than that in *Hap3*, respectively (Fig. 7B; Supplementary Table S8), indicating that promoters of *Hap1* and *Hap2* possessed higher driving activity. Sequence prediction revealed that some *cis*-elements were located at the haplotype-specific polymorphic sites (Fig. 6A), but the exact site responsible requires further confirmation.

**Fig. 8.**
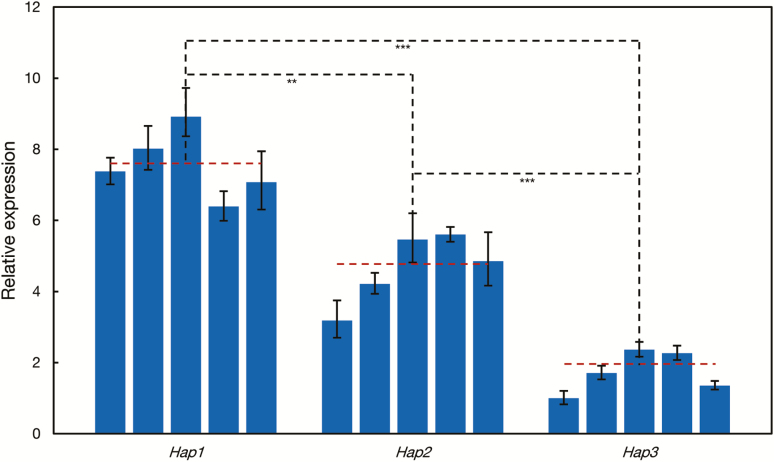
*Hap1* and *Hap2* have a higher transcription level than *Hap3* at *TaBT1-6B* in wheat collections. Dashed lines of each haplotype represent the average expression level in materials of the corresponding haplotype. **P<0.01, ***P<0.001 (*t*-test).

Considering that polymorphisms in the promoter region could influence gene transcription (Zuo and Li, 2014), we then selected five common wheat accessions of each haplotype to quantify the expression difference among *TaBT1* haplotypes. As expected, the mean expression level of *Hap1* and *Hap2* in grains at 10 DPA was 4.3 and 2.7 times higher than that of *Hap*3 (Fig. 8), generally in agreement with the GUS activities of different haplotypes. To sum up, the results from sequencing, rice transformation studies, and qRT-PCR analysis suggest that the effect on TKW is due to polymorphisms in the promoter region of *TaBT1*.

### *TaBT1* underwent selection during wheat improvement and polyploidization

**Fig. 9.**
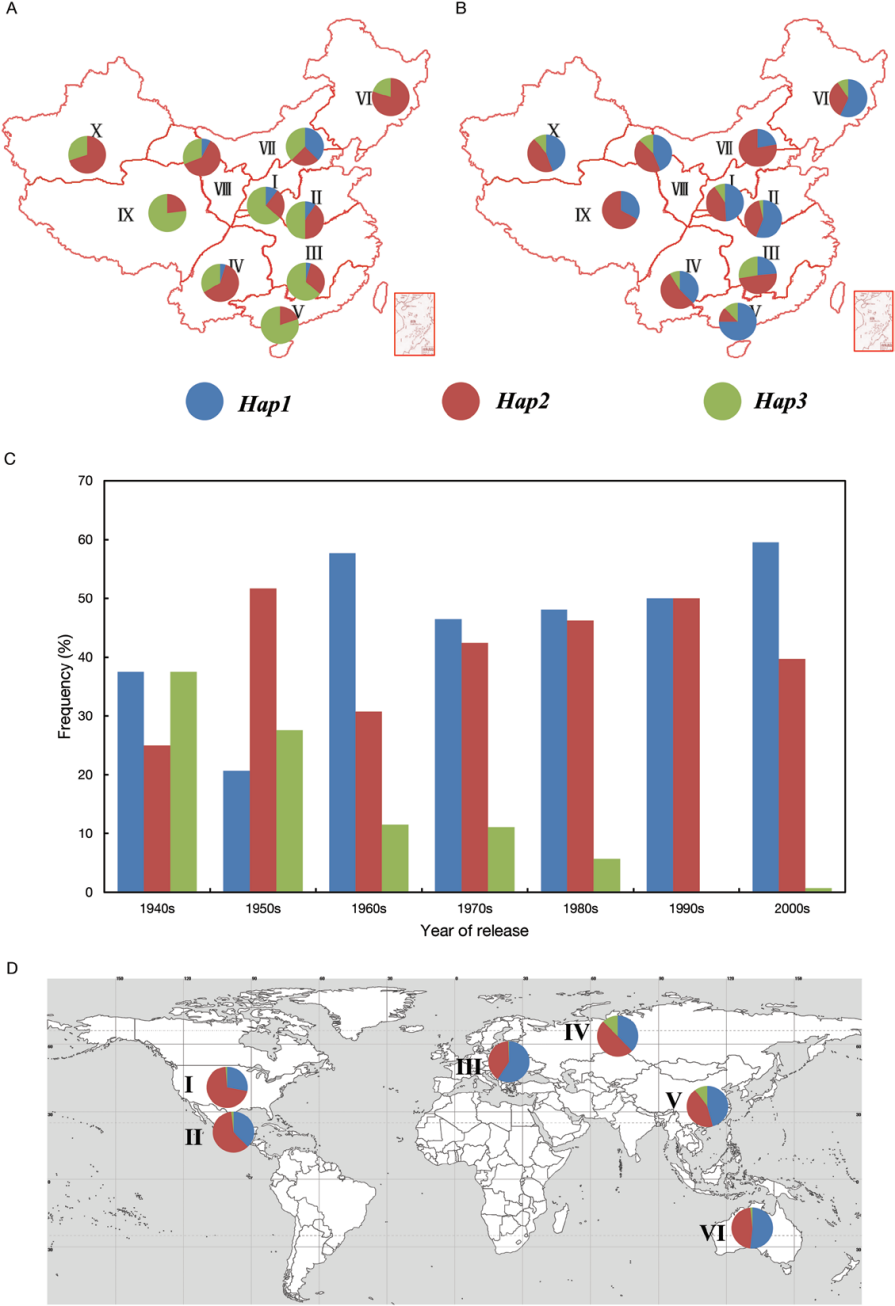
Distributions of *TaBT1-6B* haplotypes in different ecological regions and its frequency changes during the Chinese wheat breeding process. (A) 157 landraces from 10 Chinese ecological zones; (B) 348 modern cultivars from 10 Chinese ecological zones; (C) frequencies of *TaBT1-6B* haplotypes over decades in Chinese modern cultivars from the 1940s to 2000s; (D) common wheat cultivars from six major wheat production regions; I, North America; II, CIMMYT; III, Europe; IV, former USSR; V, China; VI, Australia.

Several yield-related genes have been reported to have undergone breeding selection by our group (Su *et al.*, 2011; Ma *et al.*, 2016; Hou *et al.*, 2017). To investigate whether the *TaBT1*-*6B* haplotypes were selected during the course of Chinese wheat breeding, 157 landraces from the MCC and 348 cultivars from the MC were genotyped by the two markers mentioned above. From landraces to modern cultivars, the ratio of the preferred haplotypes, *Hap1* and *Hap2*, was increased from 49.7% to 89.7% (Supplementary Table S1), and the increase was obvious in all 10 ecological wheat zones, especially in the major production zones I, II, and III (Fig. 9A–B). Further evidence showing that *Hap1* and *Hap2* underwent positive selection, while *Hap3* underwent negative selection, during Chinese wheat breeding process is provided in Fig. 9C. Their ratios in cultivars released in the other five major wheat production continents around the world are 98.7, 98.1, 99.5, 87.3, and 98.3%, respectively (Fig. 9D; Supplementary Table S5). The extremely high ratios of *Hap1* and *Hap2* in American, CIMMYT, European, and Australian wheat cultivars might be due to their longer breeding histories. The results demonstrate that *TaBT1*-*6B*-*Hap1* and -*Hap2* are dominant in global wheat cultivars and that selection pressure differs in different production regions around the world.

**Fig. 10.**
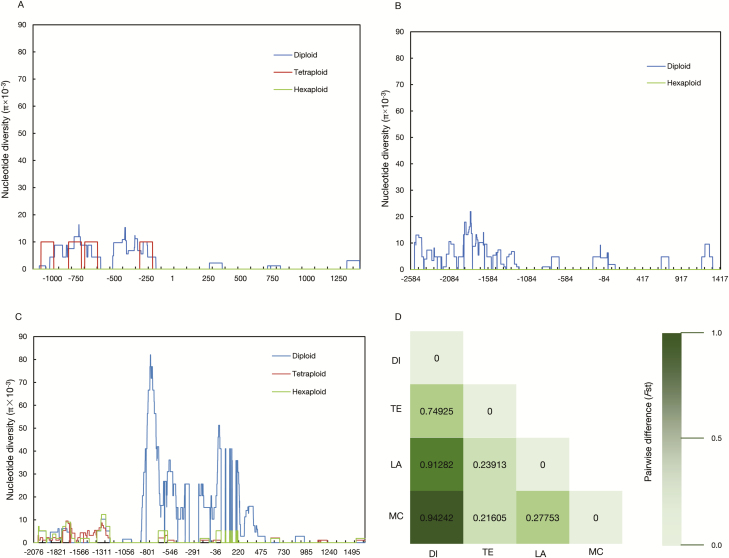
Nucleotide diversity (π) and genetic distances at *BT1* between diploid, tetraploid, and hexaploid accessions at *BT1-6A* (A), *BT1-6D* (B), and *BT1-6B* (C). (D) Genetic distances at *BT1-6B* between pairs of populations (*F*_ST_). DI, diploids; TE, tetraploids; LA, landraces; MC, modern cultivars; the color gradient presents *F*_ST_ values from dark (1.0) to pale green (0.0).

Common wheat originated from two-step polyploidization events (Kihara and Lilienfeld, 1949). To examine the evolution of *BT1* during the wheat polyploidization process, we analyzed the nucleotide diversity (π) of *BT1*-*6A*, -*6B*, and -*6D* in wheat progenitor collections. The result showed that the π value of all the three genomes declined to a different extent during polyploidization events (Fig. 10). In terms of *BT1*-*6A* and -*6D*, the decline did not reach significant levels (*P*>0.05), because they had extremely low diversity in the diploids and tetraploids (Fig. 10A–B; Table 2). However, the diversity decline at *BT1-6B* was distinct (*P*<0.05) for both the π and *F*_ST_ values between species of different ploidy (Table 2; Fig. 10D). This indicates that *BT1-6B* has undergone selection during both tetraploidization and hexaploidization.

**Table 2.** Polymorphism of *BT1* among diploid, tetraploid, and hexaploid wheat accessions

Genome	Diploid			Tetraploid			Hexaploid		
	π	Tajima’s *D*	*P*	π	Tajima’s *D*	*P*	π	Tajima’s *D*	*P*
A	0.00245	0.52410	> 0.05	0.00154	2.08033	> 0.05	0	–	–
D	0.00309	0.32379	> 0.05	–	–	–	0	–	–
B	0.00847	1.83978	> 0.05	0.00118	-1.94107	< 0.05	0.00192	2.46028	< 0.05

## Discussion

### *TaBT1* plays a vital role in starch synthesis and is significantly correlated with TKW in common wheat

BT1, which is responsible for the transmembrane transportation of ADP-Glc in endosperm starch synthesis, universally exists in cereals (Denyer *et al.*, 1996; Sikka *et al.*, 2001; Tetlow *et al.*, 2003). It affects TKW in maize and rice by regulating starch synthesis, and the mutants exhibit abnormal phenotypes (Sullivan *et al.*, 1991; Cakir *et al.*, 2016; Li *et al.*, 2017). Although *BT1* has been isolated and characterized in a variety of crops, it has not been identified in wheat and its effect on wheat yield has not been reported yet. In this study, we cloned and characterized *TaBT1* in common wheat. Expression analysis as well as the Skn-1 motif in the promoter region indicate that *TaBT1* has a grain-specific expression pattern. *TaBT1* homoeologous genes were highly expressed in developing grains at 10 and 15 DPA, the period during which starch is being synthesized in the endosperm (Fig. 2), similarly to that of rice *OsBT1-1* (Toyota *et al.*, 2006). We may also infer that amyloplast formation has almost been completed by 15–20 DPA (Fig. 2B), but starch synthesis can be detected even at 35–40 DPA in wheat endosperm in major Chinese production regions.

Previous studies indicate that the formation of A-type starch granules mainly initiates at 4–15 DPA, while that of B-type granules generally starts at 15 DPA and lasts until the grain matures (Darlington *et al.*, 2000; Peng *et al.*, 2000). In this study, the expression of *TaBT1* in transgenic RNAi lines was knocked down, which may subsequently affect the formation of starch granules and total starch content (Figs 4, 5). In addition, the transgenic wheat exhibited a significant decrease in TKW, compared with the wild type (Fig. 3E). All of the above results indicate that *TaBT1* plays a vital role in starch synthesis and is significantly correlated with TKW in common wheat.

### Natural variation in the promoter region of *TaBT1*-*6B* influences its activity

Since *TaBT1* has a significant effect on TKW, we then investigated its diversity in common wheat in an attempt to find elite allelic variations. Three haplotypes at *TaBT1*-*6B* were formed by 24 polymorphic sites, and most variations were located in the promoter region. Previous studies have shown that polymorphisms in gene promoter regions could give rise to variation in yield-related traits by affecting binding elements and subsequent expression (Li *et al.*, 2011; Qin *et al.*, 2014; Zuo and Li, 2014). For example, variation in the promoter of *OsGS5* leads to expression level changes and grain width variations (Li *et al.*, 2011). In this study, some *cis*-elements were previously found to be haplotype specific (Fig. 6A). For instance, an element named PRECONSCRHSP70A, involved in the expression of *HSP70A* under the induction of Mg-protoporphyrin IX and light (von Gromoff *et al.*, 2006), was found at base pair –1271 of *Hap1*. Moreover, an ASF1 motif involved in transcriptional activation of several genes is present at base pair –1813 of *Hap3* (Després *et al.*, 2003). The influence of these *cis*-elements on the expression of *TaBT1* needs to be further investigated. Both GUS activities in transgenic rice grains with exogenous promoters and expression of *TaBT1*-*6B* in varieties of the three haplotypes indicate that *Hap1* and *Hap2* possess higher promoter activity than *Hap3* (Figs 7, 8; Supplementary Table S8). Moreover, association analysis between haplotypes and yield-related phenotypes in the MCC and MC, combined with TKW differences between *Hap1* and *Hap3* in introgression lines (Table 1; Supplementary Table S2), further indicate that *Hap1* and *Hap2* were favorable haplotypes with a higher TKW. Thus, polymorphisms in the *TaBT1-6B* promoter region influence its driving activity and further affect TKW in wheat.

### Potential application of molecular markers in wheat breeding

Genetic diversity analysis of functional genes is important for understanding the genetic background of phenotypic variation (Xu *et al.*, 2014). Abundant genetic variation in wheat enables breeders to create better gene combinations and select superior varieties to meet local breeding objectives. Marker-assisted selection (MAS) provides an efficient method for genetic improvement of crops. Many functional markers associated with important agronomic traits, such as grain weight (Ma *et al.*, 2016; Hou *et al.*, 2017), plant height (Li *et al.*, 2012), photoperiod response (Beales *et al.*, 2007), disease resistance (Li *et al.*, 2014), and end-use quality (Sun *et al.*, 2005; He *et al.*, 2007), can be widely used in MAS (William *et al.*, 2007; Liu *et al.*, 2012). Based on variations in the promoter region of *TaBT1*-*6B*, the functional markers *CAPS-1664* and *InDel-2029* were developed to distinguish the three haplotypes in germplasm from China and five other global major wheat production regions (Fig. 9). These markers are co-dominant and can be easily implemented in the laboratory. Their potential value for selection of TKW was validated by association analysis; *TaBT1*-*6B*-*Hap1* and -*Hap2* identified with the two markers were correlated with higher TKW. Although the frequency of *Hap1* and *Hap2* was high enough among MC globally, further increases are still feasible in regions where *Hap1* occurs at relatively lower frequencies. In addition, functional markers related to higher TKW so far reported are available for *TaSus2*-*2A*-*HapA*, *TaGS5*-*3A*-*T*, and *TaAGP*-*S1*-*7A*-*HapI* (Hou *et al.*, 2014, 2017; Ma *et al.*, 2016); pyramiding preferred alleles of these genes with MAS will be instrumental in wheat breeding by enhancing additive genetic variation.

### *TaBT1* has undergone selection during wheat polyploidization and improvement

Although domestication and modern breeding contribute to the development of different ecotypes and cultivars, genetic diversity of genes controlling agronomic traits has also been narrowed down (Haudry *et al.*, 2007). Several yield-related genes in wheat have undergone genetic diversity reduction during polyploidization, such as the sucrose synthase gene (*TaSUS1*), the grain size gene (*TaGS5*), and the ubiquitin E3 ligase gene (*TaGW2*) (Su *et al.*, 2011; Hou *et al.*, 2014; Ma *et al.*, 2016; Qin *et al.*, 2017). In this study, the genetic diversity of *BT1* differs among the three wheat genomes, with the highest identified in the B genome, followed by the D and A genomes; this is in general accord with previous studies (Gao *et al.*, 2017; Hao *et al.*, 2017). The higher diversity in the B genome may be due to the cross-pollination characteristics of *Aegilops speltoides*, the most likely donor of the B genome (Zohary and Imber, 1963; Shewry, 2009). However, the low diversity in the D genome was due to the bottleneck when common wheat arose from the extremely few hybridizations of tetraploid wheat with *Aegilops tauschii* ~10 000 years ago (Lupton, 1987; Petersen *et al.*, 2006). Additionally, the A genome underwent higher selection pressure due to it carrying more agronomic trait-related genes (Peng *et al.*, 2003; Marcussen *et al.*, 2014; Hao *et al.*, 2017). The extreme low diversity of *BT1-6A* and *BT1-6D* indirectly reflects the crucial role of this gene for propagation of these species (Fig. 10; Table 2).

TKW has been continually improved during evolution from diploid to tetraploid and to the widely cultivated hexaploid common wheat. This may be due to the accumulation of preferred haplotypes associated with high TKW. For example, the frequencies of *TaBT1*-*6B*-*Hap1* and -*Hap2* increased from Chinese landraces to MCs, indicating that the two haplotypes were positively selected during modern wheat breeding. Moreover, *Hap1* and *Hap2* were present at very high frequencies in varieties from five other global major wheat production regions. This suggests that selection for preferred haplotypes with high TKW was global, while frequencies of *Hap1* could still be beneficially elevated.

## Supplementary data

Supplementary data are available at *JXB* online.

Table S1. Accessions of hexaploid wheat in the mini-core collection (MCC) and modern cultivars (MCs) in China.

Table S2. Thousand kernel weight data for introgression lines derived from Fumai/Handan 6172 (BC_3_F_7_).

Table S3. Fifteen common wheat cultivars used for expression analysis among *TaBT1-6B* haplotypes.

Table S4. Progenitor accessions used in this study.

Table S5. Accessions of common wheat from five non-Chinese major wheat production regions.

Table S6. Primers used in this study.

Table S7. Thirty-six common wheat cultivars used for haplotype analysis of *TaBT1*.

Table S8. GUS activities in transgenic rice grains.

Fig. S1. Circadian expression of *TaBT1*-*6A*/*6B*/*6D*.

Fig. S2. Effective tiller number (ETN), spike number (SN), and grain number (GN) in *TaBT1* transgenic RNAi lines.

Fig. S3. Southern blot detection of *bar* genes for the identification of transgenic wheat plants in the T_2_ generation.

Fig. S4. Relative expression of three homoeologous genes in *TaBT1*-RNAi transgenic wheat.

Supplementary Figures S1-S4

Supplementary Table S1-S8

## Abbreviations:

ETN: effective tiller number
GN: grain number per spike
GUS: β-glucuronidase
KL: kernel length
KT: kernel thickness
KW: kernel width
MAS: marker-assisted selection
MC: modern cultivars
MCC: mini-core collection
SN: spikelet number per spike
SNP: single nucleotide polymorphism
TKW: thousand kernel weight

## References

[CIT0001] Barron C, Surget A, Rouau X 2007 Relative amounts of tissues in mature wheat (*Triticum aestivum* L.) grain and their carbohydrate and phenolic acid composition. Journal of Cereal Science 45, 88-96.

[CIT0002] Beales J, Turner A, Griffiths S, Snape JW, Laurie DA 2007 *A pseudo-response regulator* is misexpressed in the photoperiod insensitive *Ppd*-*D1a* mutant of wheat (*Triticum aestivum* L.). Theoretical and Applied Genetics 115, 721–733. 17634915 10.1007/s00122-007-0603-4

[CIT0003] Beckles DM, Smith AM, ap Rees T 2001 A cytosolic ADP-glucose pyrophosphorylase is a feature of graminaceous endosperms, but not of other starch-storing organs. Plant Physiology 125, 818–827. 11161039 10.1104/pp.125.2.818 PMC64883

[CIT0004] Bowsher CG, Scrase-Field EF, Esposito S, Emes MJ, Tetlow IJ 2007 Characterization of ADP-glucose transport across the cereal endosperm amyloplast envelope. Journal of Experimental Botany 58, 1321–1332. 17301030 10.1093/jxb/erl297

[CIT0005] Bradford MM 1976 A rapid and sensitive method for the quantitation of microgram quantities of protein utilizing the principle of protein–dye binding. Analytical Biochemistry 72, 248–254. 942051 10.1016/0003-2697(76)90527-3

[CIT0006] Burton RA, Johnson PE, Beckles DM, Fincher GB, Jenner HL, Naldrett MJ, Denyer K 2002 Characterization of the genes encoding the cytosolic and plastidial forms of ADP-glucose pyrophosphorylase in wheat endosperm. Plant Physiology 130, 1464–1475. 12428011 10.1104/pp.010363 PMC166665

[CIT0007] Cakir B, Shiraishi S, Tuncel A, et al 2016 Analysis of the rice ADP-Glucose Transporter (*OsBT1*) indicates the presence of regulatory processes in the amyloplast stroma that control ADP-glucose flux into starch. Plant Physiology 170, 1271–1283. 26754668 10.1104/pp.15.01911 PMC4775147

[CIT0008] Darlington HF, Tecsi L, Harris N, Griggs DL, Cantrell IC, Shewry PR 2000 Starch granule associated proteins in barley and wheat. Journal of Cereal Science 32, 21–29.

[CIT0009] Denyer K, Dunlap F, Thorbjørnsen T, Keeling P, Smith AM 1996 The major form of ADP-glucose pyrophosphorylase in maize endosperm is extra-plastidial. Plant Physiology 112, 779–785. 8883389 10.1104/pp.112.2.779 PMC158002

[CIT0010] Deol KK, Mukherjee S, Gao F, Brûlé-Babel A, Stasolla C, Ayele BT 2013 Identification and characterization of the three homeologues of a new sucrose transporter in hexaploid wheat (*Triticum aestivum* L.). BMC Plant Biology 13, 181. 24237613 10.1186/1471-2229-13-181 PMC4225610

[CIT0011] Després C, Chubak C, Rochon A, Clark R, Bethune T, Desveaux D, Fobert PR 2003 The Arabidopsis NPR1 disease resistance protein is a novel cofactor that confers redox regulation of DNA binding activity to the basic domain/leucine zipper transcription factor TGA1. The Plant Cell 15, 2181–2191. 12953119 10.1105/tpc.012849 PMC181339

[CIT0012] Doyle JJ 1987 A rapid DNA isolation procedure for small quantities of fresh leaf tissue. Phytochem Bull 19, 11–15.

[CIT0013] Gao L, Zhao G, Huang D, Jia J 2017 Candidate loci involved in domestication and improvement detected by a published 90K wheat SNP array. Scientific Reports 7, 44530. 28327671 10.1038/srep44530 PMC5361097

[CIT0014] Haferkamp I 2007 The diverse members of the mitochondrial carrier family in plants. FEBS Letters 581, 2375–2379. 17321523 10.1016/j.febslet.2007.02.020

[CIT0015] Hänsch R, Koprek T, Mendel RR, Schulze J 1995 An improved protocol for eliminating endogenous β-glucuronidase background in barley. Plant Science 105, 63–69.

[CIT0016] Hao C, Wang L, Ge H, Dong Y, Zhang X 2011 Genetic diversity and linkage disequilibrium in Chinese bread wheat (*Triticum aestivum* L.) revealed by SSR markers. PLoS One 6, e17279. 21365016 10.1371/journal.pone.0017279 PMC3041829

[CIT0017] Hao C, Wang Y, Chao S, Li T, Liu H, Wang L, Zhang X 2017 The iSelect 9 K SNP analysis revealed polyploidization induced revolutionary changes and intense human selection causing strong haplotype blocks in wheat. Scientific Reports 7, 41247. 28134278 10.1038/srep41247 PMC5278348

[CIT0018] Haudry A, Cenci A, Ravel C, et al 2007 Grinding up wheat: a massive loss of nucleotide diversity since domestication. Molecular Biology and Evolution 24, 1506–1517. 17443011 10.1093/molbev/msm077

[CIT0019] He XY, He ZH, Zhang LP, Sun DJ, Morris CF, Fuerst EP, Xia XC 2007 Allelic variation of polyphenol oxidase (PPO) genes located on chromosomes 2A and 2D and development of functional markers for the PPO genes in common wheat. Theoretical and Applied Genetics 115, 47–58. 17426955 10.1007/s00122-007-0539-8

[CIT0020] Hiei Y, Ohta S, Komari T, Kumashiro T 1994 Efficient transformation of rice (*Oryza sativa* L.) mediated by Agrobacterium and sequence analysis of the boundaries of the T-DNA. The Plant Journal 6, 271–282. 7920717 10.1046/j.1365-313x.1994.6020271.x

[CIT0021] Hou J, Jiang Q, Hao C, Wang Y, Zhang H, Zhang X 2014 Global selection on sucrose synthase haplotypes during a century of wheat breeding. Plant Physiology 164, 1918–1929. 24402050 10.1104/pp.113.232454 PMC3982753

[CIT0022] Hou J, Li T, Wang Y, Hao C, Liu H, Zhang X 2017 ADP-glucose pyrophosphorylase genes, associated with kernel weight, underwent selection during wheat domestication and breeding. Plant Biotechnology Journal 15, 1533–1543. 28371241 10.1111/pbi.12735 PMC5698054

[CIT0023] International Wheat Genome Sequencing Consortium. 2014 A chromosome-based draft sequence of the hexaploid bread wheat (*Triticum aestivum*) genome. Science 345, 1251788. 25035500 10.1126/science.1251788

[CIT0024] Khush GS 2013 Strategies for increasing the yield potential of cereals: case of rice as an example. Plant Breeding 132, 433–436.

[CIT0025] Kihara H, Lilienfeld F 1949 A new synthesized 6×‐wheat. Hereditas 35, 307–319.

[CIT0026] Kirchberger S, Leroch M, Huynen MA, Wahl M, Neuhaus HE, Tjaden J 2007 Molecular and biochemical analysis of the plastidic ADP-glucose transporter (*ZmBT1*) from *Zea mays*. Journal of Biological Chemistry 282, 22481–22491. 17562699 10.1074/jbc.M702484200

[CIT0027] Kirchberger S, Tjaden J, Neuhaus HE 2008 Characterization of the *Arabidopsis* Brittle1 transport protein and impact of reduced activity on plant metabolism. The Plant Journal 56, 51–63. 18564385 10.1111/j.1365-313X.2008.03583.x

[CIT0028] Kosugi S, Ohashi Y, Nakajima K 1990 An improved assay for β-glucuronidase in transformed cells: methanol almost completely suppresses a putative endogenous β-glucuronidase activity. Plant Science 70, 133–140.

[CIT0029] Kumar R, Mukherjee S, Ayele BT 2018 Molecular aspects of sucrose transport and its metabolism to starch during seed development in wheat: a comprehensive review. Biotechnology Advances 36, 954–967. 29499342 10.1016/j.biotechadv.2018.02.015

[CIT0030] Leroch M, Kirchberger S, Haferkamp I, Wahl M, Neuhaus HE, Tjaden J 2005 Identification and characterization of a novel plastidic adenine nucleotide uniporter from *Solanum tuberosum*. Journal of Biological Chemistry 280, 17992–18000. 15737999 10.1074/jbc.M412462200

[CIT0031] Li S, Wei X, Ren Y, Qiu J, Jiao G, Guo X, Tang S, Wan J, Hu P 2017 *OsBT1* encodes an ADP-glucose transporter involved in starch synthesis and compound granule formation in rice endosperm. Scientific Reports 7, 40124. 28054650 10.1038/srep40124 PMC5215005

[CIT0032] Li Y, Fan C, Xing Y, et al 2011 Natural variation in *GS5* plays an important role in regulating grain size and yield in rice. Nature Genetics 43, 1266–1269. 22019783 10.1038/ng.977

[CIT0033] Li Y, Xiao J, Wu J, et al 2012 A tandem segmental duplication (TSD) in green revolution gene *Rht-D1b* region underlies plant height variation. New Phytologist 196, 282–291. 22849513 10.1111/j.1469-8137.2012.04243.x

[CIT0034] Li ZF, Lan CX, He ZH, Singh RP, Rosewarne GM, Chen XM, Xia XC 2014 Overview and application of QTL for adult plant resistance to leaf rust and powdery mildew in wheat. Crop Science 54, 1907–1925.

[CIT0035] Liu KC, Boyer CD, Shannon JC 1992 Carbohydrate transfer into isolated maize (*Zea mays* L.) amyloplasts. Plant Physiology 99 (Suppl), 39.

[CIT0036] Liu Y, He Z, Appels R, Xia X 2012 Functional markers in wheat: current status and future prospects. Theoretical and Applied Genetics 125, 1–10. 22366867 10.1007/s00122-012-1829-3

[CIT0037] Lupton FGH, ed. 1987 Wheat breeding: its scientific basis. London: Chapman and Hall Ltd.

[CIT0038] Ma L, Li T, Hao C, Wang Y, Chen X, Zhang X 2016 *TaGS5-3A*, a grain size gene selected during wheat improvement for larger kernel and yield. Plant Biotechnology Journal 14, 1269–1280. 26480952 10.1111/pbi.12492 PMC11389196

[CIT0039] Mangelsdorf PC 1926 Genetics and morphology of some endosperm characters in maize. New Haven, CT: Connecticut Agricultural Experiment Station.

[CIT0040] Mao X, Jia D, Li A, Zhang H, Tian S, Zhang X, Jia J, Jing R 2011 Transgenic expression of *TaMYB2A* confers enhanced tolerance to multiple abiotic stresses in *Arabidopsis*. Functional & Integrative Genomics 11, 445–465. 21472467 10.1007/s10142-011-0218-3

[CIT0041] Marcussen T, Sandve SR, Heier L, et al 2014 Ancient hybridizations among the ancestral genomes of bread wheat. Science 345, 1250092. 25035499 10.1126/science.1250092

[CIT0042] Millar AH, Heazlewood JL 2003 Genomic and proteomic analysis of mitochondrial carrier proteins in *Arabidopsis*. Plant Physiology 131, 443–453. 12586869 10.1104/pp.009985 PMC166821

[CIT0043] Neuhaus HE, Emes MJ 2000 Nonphotosynthetic metabolism in plastids. Annual Review of Plant Physiology and Plant Molecular Biology 51, 111–140. 10.1146/annurev.arplant.51.1.111 15012188

[CIT0044] Palmieri F, Monné M 2016 Discoveries, metabolic roles and diseases of mitochondrial carriers: a review. Biochimica et Biophysica Acta 1863, 2362–2378. 26968366 10.1016/j.bbamcr.2016.03.007

[CIT0045] Palmieri L, Arrigoni R, Blanco E, Carrari F, Zanor MI, Studart-Guimaraes C, Fernie AR, Palmieri F 2006 Molecular identification of an *Arabidopsis* S-adenosylmethionine transporter. Analysis of organ distribution, bacterial expression, reconstitution into liposomes, and functional characterization. Plant Physiology 142, 855–865. 16950860 10.1104/pp.106.086975 PMC1630753

[CIT0046] Peng JH, Ronin Y, Fahima T, Röder MS, Li YC, Nevo E, Korol A 2003 Domestication quantitative trait loci in *Triticum dicoccoides*, the progenitor of wheat. Proceedings of the National Academy of Sciences, USA 100, 2489–2494. 10.1073/pnas.252763199 PMC151368 12604784

[CIT0047] Peng M, Gao M, Båga M, Hucl P, Chibbar RN 2000 Starch-branching enzymes preferentially associated with A-type starch granules in wheat endosperm. Plant Physiology 124, 265–272. 10982441 10.1104/pp.124.1.265 PMC59141

[CIT0048] Petersen G, Seberg O, Yde M, Berthelsen K 2006 Phylogenetic relationships of *Triticum* and *Aegilops* and evidence for the origin of the A, B, and D genomes of common wheat (*Triticum aestivum*). Molecular Phylogenetics and Evolution 39, 70–82. 16504543 10.1016/j.ympev.2006.01.023

[CIT0049] Qin L, Hao C, Hou J, Wang Y, Li T, Wang L, Ma Z, Zhang X 2014 Homologous haplotypes, expression, genetic effects and geographic distribution of the wheat yield gene *TaGW2*. BMC Plant Biology 14, 107. 24766773 10.1186/1471-2229-14-107 PMC4021350

[CIT0050] Qin L, Zhao J, Li T, Hou J, Zhang X, Hao C 2017 *TaGW2*, a good reflection of wheat polyploidization and evolution. Frontiers in Plant Science 8, 318. 28326096 10.3389/fpls.2017.00318 PMC5339256

[CIT0051] Sambrook J, Fritsch EF, Maniatis T 1989 Molecular cloning: a laboratory manual, 2nd edn. Cold Spring Harbor, NY: Cold Spring Harbor Laboratory Press.

[CIT0052] Shannon JC, Pien FM, Cao H, Liu KC 1998 Brittle-1, an adenylate translocator, facilitates transfer of extraplastidial synthesized ADP–glucose into amyloplasts of maize endosperms. Plant Physiology 117, 1235–1252. 9701580 10.1104/pp.117.4.1235 PMC34888

[CIT0053] Shannon JC, Pien FM, Liu KC 1996 Nucleotides and nucleotide sugars in developing maize endosperms. Synthesis of ADP-glucose in *brittle*-1. Plant Physiology 110, 835–843. 12226222 10.1104/pp.110.3.835 PMC157783

[CIT0054] Shewry PR 2009 Wheat. Journal of Experimental Botany 60, 1537–1553. 19386614 10.1093/jxb/erp058

[CIT0055] Sikka VK, Choi SB, Kavakli IH, Sakulsingharoj C, Gupta S, Ito H, Okita TW 2001 Subcellular compartmentation and allosteric regulation of the rice endosperm ADPglucose pyrophosphorylase. Plant Science 161, 461–468.

[CIT0056] Soliman A, Ayele BT, Daayf F 2014 Biochemical and molecular characterization of barley plastidial ADP-glucose transporter (*HvBT1*). PLoS One 9, e98524. 24892865 10.1371/journal.pone.0098524 PMC4043945

[CIT0057] Su Z, Hao C, Wang L, Dong Y, Zhang X 2011 Identification and development of a functional marker of *TaGW2* associated with grain weight in bread wheat (*Triticum aestivum* L.). Theoretical and Applied Genetics 122, 211–223. 20838758 10.1007/s00122-010-1437-z

[CIT0058] Sullivan TD, Strelow LI, Illingworth CA, Phillips RL, Nelson OE Jr 1991 Analysis of maize brittle-1 alleles and a defective suppressor-mutator-induced mutable allele. The Plant Cell 3, 1337–1348. 1668652 10.1105/tpc.3.12.1337 PMC160096

[CIT0059] Sun DJ, He ZH, Xia XC, Zhang LP, Morris CF, Appels R, Ma WJ 2005 A novel STS marker for polyphenol oxidase activity in bread wheat. Molecular Breeding 16, 209–218.

[CIT0060] Tetlow IJ, Davies EJ, Vardy KA, Bowsher CG, Burrell MM, Emes MJ 2003 Subcellular localization of ADPglucose pyrophosphorylase in developing wheat endosperm and analysis of the properties of a plastidial isoform. Journal of Experimental Botany 54, 715–725. 12554715 10.1093/jxb/erg088

[CIT0061] Thorbjørnsen T, Villand P, Kleczkowski LA, Olsen OA 1996 A single gene encodes two different transcripts for the ADP-glucose pyrophosphorylase small subunit from barley (*Hordeum vulgare*). Biochemical Journal 313, 149–154. 8546676 10.1042/bj3130149 PMC1216875

[CIT0062] Tobias RB, Boyer CD, Shannon JC 1992 Alterations in carbohydrate intermediates in the endosperm of starch-deficient maize (*Zea mays* L.) genotypes. Plant Physiology 99, 146–152. 16668842 10.1104/pp.99.1.146 PMC1080419

[CIT0063] Toyota K, Tamura M, Ohdan T, Nakamura Y 2006 Expression profiling of starch metabolism-related plastidic translocator genes in rice. Planta 223, 248–257. 16362329 10.1007/s00425-005-0128-5

[CIT0064] von Gromoff ED, Schroda M, Oster U, Beck CF 2006 Identification of a plastid response element that acts as an enhancer within the Chlamydomonas HSP70A promoter. Nucleic Acids Research 34, 4767–4779. 16971458 10.1093/nar/gkl602 PMC1635268

[CIT0065] Wang K, Liu H, Du L, Ye X 2017 Generation of marker-free transgenic hexaploid wheat via an *Agrobacterium*-mediated co-transformation strategy in commercial Chinese wheat varieties. Plant Biotechnology Journal 15, 614–623. 27862820 10.1111/pbi.12660 PMC5399001

[CIT0066] Wang L, Ge H, Hao C, Dong Y, Zhang X 2012 Identifying loci influencing 1,000-kernel weight in wheat by microsatellite screening for evidence of selection during breeding. PLoS One 7, e29432. 22328917 10.1371/journal.pone.0029432 PMC3273457

[CIT0067] William HM, Trethowan R, Crosby-Galvan EM 2007 Wheat breeding assisted by markers: CIMMYT’s experience. Euphytica 157, 307–319.

[CIT0068] Xu S, Yang Z, Zhang E, Jiang Y, Pan L, Chen Q, Xie Z, Xu C 2014 Nucleotide diversity of maize *ZmBT1* gene and association with starch physicochemical properties. PLoS One 9, e103627. 25084007 10.1371/journal.pone.0103627 PMC4118901

[CIT0069] Zhang D, Hao C, Wang L, Zhang X 2012 Identifying loci influencing grain number by microsatellite screening in bread wheat (*Triticum aestivum* L.). Planta 236, 1507–1517. 22820969 10.1007/s00425-012-1708-9

[CIT0070] Zheng J, Liu H, Wang Y, Wang L, Chang X, Jing R, Hao C, Zhang X 2014 TEF-7A, a transcript elongation factor gene, influences yield-related traits in bread wheat (*Triticum aestivum* L.). Journal of Experimental Botany 65, 5351–5365. 25056774 10.1093/jxb/eru306 PMC4157721

[CIT0071] Zheng TC, Zhang XK, Yin GH, Wang LN, Han YL, Chen L, Huang F, Tang JW, Xia XC, He ZH 2011 Genetic gains in grain yield, net photosynthesis and stomatal conductance achieved in Henan Province of China between 1981 and 2008. Field Crop Research 122, 225–233.

[CIT0072] Zohary D, Imber D 1963 Genetic dimorphism in fruit types in *Aegilops speltoides*. Heredity 18, 223–231.

[CIT0073] Zuo J, Li J 2014 Molecular genetic dissection of quantitative trait loci regulating rice grain size. Annual Review of Genetics 48, 99–118. 10.1146/annurev-genet-120213-092138 25149369

[CIT0074] Zuo JR, Li JY 2013 Molecular dissection of complex agronomic traits of rice: a team effort by Chinese scientists in recent years. National Science Review 1, 253–276.

