# Supplementary material for: TaBT1, affecting starch synthesis and thousand kernel weight, underwent strong selection during wheat improvement
Source: J Exp Bot. 2019 Feb 7;70(5):1497–511. doi: 10.1093/jxb/erz032 (PMC6411380; doi:10.1093/jxb/erz032)
Supplement: Supplementary Figures S1-S4 [file erz032_suppl_supplementary_figures-s1-s4.pdf]

TaBT1 affecting starch synthesis and thousand kernel weight, underwent strong selection during wheat improvement

Yamei Wang[1], Jian Hou[1], Hong Liu[1,2], Tian Li[1], Ke Wang[1], Chenyang Hao[1], Hongxia Liu[1], Xueyong Zhang[1†]

Supplementary figure legends

Fig. S1. Circadian expression of *TaBT1-6A*/*6B*/*6D*. Expression of *TaBT1-6A* in grains at 0 h was assumed to be 1. The white and black rectangles represent light and dark periods, respectively.

Fig. S2. Effective tiller number (ETN), spike number (SN) and grain number (GN) in *TaBT1* transgenic RNAi lines.

Fig. S3. Southern blot detection of *bar* genes for the identification of transgenic wheat plants in the T_2 generation. 1-6 denote different individual plants. 1, RNAi-L1, bar+; 2, RNAi-L2, bar+; 3, RNAi-L3, bar+; 4, WT, bar-, 5, Neg-RNAi-1, bar-; M, 15000 bp DNA marker.

Fig. S4. Relative expression of three homoeologous genes in *TaBT1*-RNAi transgenic wheat. Expression of *TaBT1-6A* in grains at 10 DPA was assumed to be 1.

Figure S1

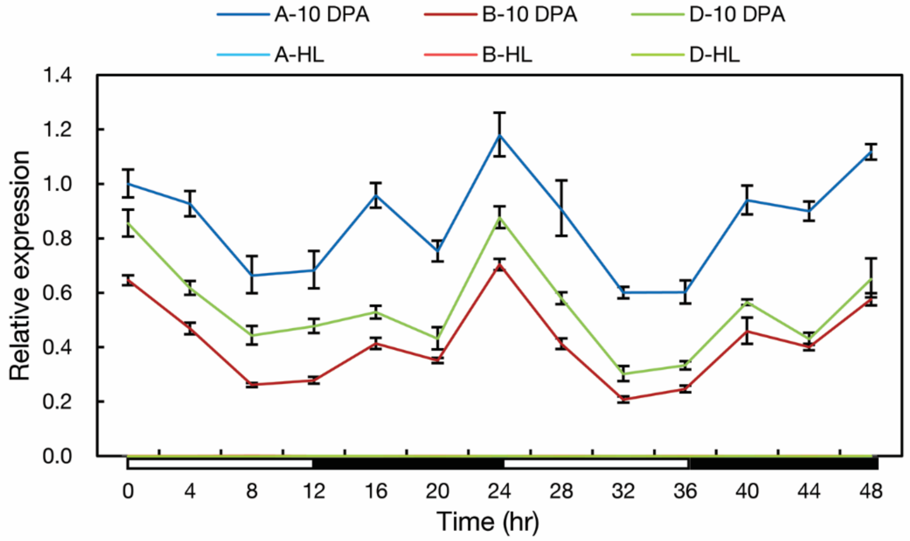
A-10 DPA
B-10 DPA
D-10 DPA
A-HL
B-HL
D-HL
1.4
1.2
1.0
0.8
0.6
0.4
0.2
0.0
Relative expression
0
4
8
12
16
20
24
28
32
36
40
44
48
Time (hr)

Figure S2

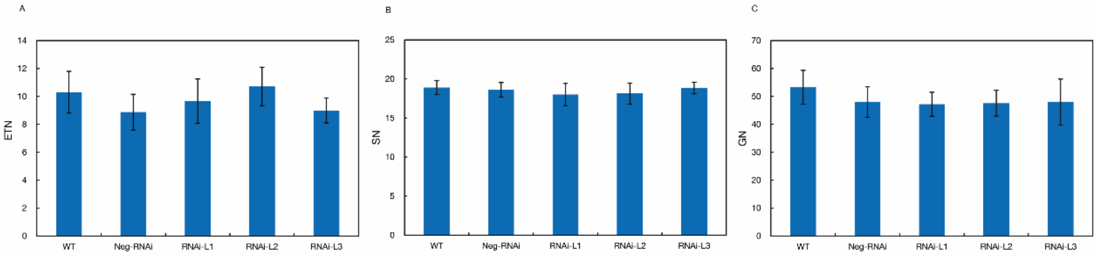
A
ETN
14
12
10
8
6
4
2
0
WT
Neg-RNAi
RNAi-L1
RNAi-L2
RNAi-L3
B
SN
25
20
15
10
5
0
WT
Neg-RNAi
RNAi-L1
RNAi-L2
RNAi-L3
C
GN
70
60
50
40
30
20
10
0
WT
Neg-RNAi
RNAi-L1
RNAi-L2
RNAi-L3

Figure S3

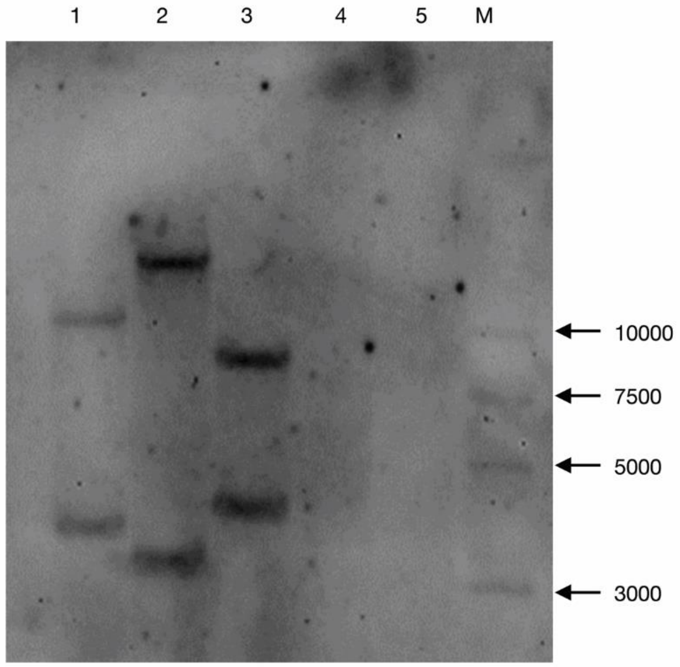

Figure S4

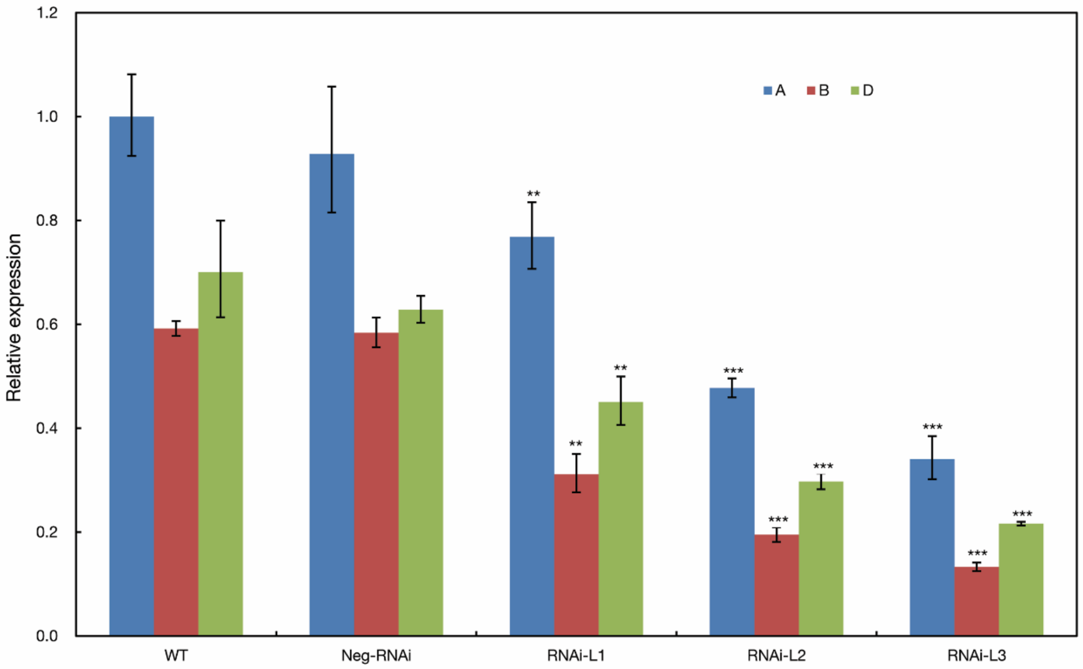

1.2
1.0
0.8
0.6
0.4
0.2
0.0
Relative expression
A
B
D
**
**
**
***
***
***
***
***
***
WT
Neg-RNAi
RNAi-L1
RNAi-L2
RNAi-L3
